# Depletion of CD8^+^ T cells from vaccinated goats does not affect protection from challenge with wild‐type peste des petits ruminants virus

**DOI:** 10.1111/tbed.13936

**Published:** 2020-12-20

**Authors:** Michael D. Baron, Sophia Hodgson, Katy Moffat, Mehnaz Qureshi, Simon P. Graham, Karin E. Darpel

**Affiliations:** ^1^ The Pirbright Institute Pirbright UK; ^2^ School of Veterinary Medicine University of Surrey Guildford UK

**Keywords:** CD8^+^ T cells, correlates of protection, immune response, Peste des petits ruminants, vaccine

## Abstract

Peste des petits ruminants (PPR) is a severe disease of goats and sheep that is widespread in Africa, the Middle East and Asia. The disease is caused by peste des petits ruminants virus (PPRV); cell culture‐attenuated strains of PPRV have been shown, both experimentally and by extensive use in the field, to be effective vaccines and are widely used. We have previously demonstrated that these vaccines elicit both serological (PPRV‐specific antibody) and cell‐based (PPRV‐specific CD4^+^ and CD8^+^ T cells) immune responses. However, it is not known which of these responses are required for protection from PPRV, information that would be useful in the evaluation of new vaccines that are being developed to provide the capability to differentiate infected and vaccinated animals (DIVA capability). To begin to address this issue, we have used a complement‐fixing monoclonal antibody recognizing caprine CD8 to deplete >99.9% of circulating CD8^+^ T cells from vaccinated goats. Animals were then infected with wild‐type PPRV. Despite the absence of the CD8^+^ T‐cell component of the vaccine‐induced immune response, the vaccinated animals were almost fully protected, showing no pyrexia or viraemia, and almost no clinical signs. These data suggest that a virus‐specific CD8^+^ T‐cell response is not critical for protection against PPRV and that virus‐specific antibody and/or CD4^+^ T cells are the main mediators of protection. We have also shown that the leucopenia caused by infection with wild‐type PPRV affects all major classes of circulating leucocytes.

## 1 INTRODUCTION

The members of the genus *Morbillivirus*, family Paramyxoviridae, are a closely related group of viruses that cause disease in distinct groups of mammals. Measles virus (MeV) is the type virus of the genus and causes disease in humans, while rinderpest virus (RPV) was historically a much feared pathogen of cattle and buffalo, although the disease is now eradicated in nature (FAO & OIE, 2011), the second viral disease to be eradicated. The genus also includes canine distemper virus (CDV), a common pathogen of domestic and wild dogs which has also been found in mustelids such as mink and in the freshwater seals of Lake Baikal, and peste des petits ruminants virus (PPRV) (species name *Small ruminant morbillivirus*) which causes a severe disease (PPR) in small ruminants, particularly sheep and goats but also several species of wild antelope (Baron et al., 2016), including the endangered Siberian saiga (Aguilar et al., 2018). Attenuated forms of several morbilliviruses have been derived by serial passage in cell culture and/or eggs (e.g. the Plowright strain of RPV [Plowright & Ferris, 1962], the Edmonston strain of MeV [Enders et al., 1960] and the Nigeria/75 strain of PPRV [Diallo et al., 1989]), and these attenuated viruses have proven to be safe and effective vaccines which have allowed the eradication of rinderpest (FAO & OIE, 2011) and a major worldwide reduction in cases of measles (Moss, 2017). PPR is now the target of a global control and eradication programme co‐ordinated by the Food and Agriculture Organisation of the United Nations (FAO) and the World Organisation for Animal Health (OIE) (FAO & OIE, 2015).

While experience has shown that these vaccines have broad spectrum efficacy, the exact nature of the protective immune response is not known. We have previously shown that vaccination with either of two live attenuated PPRV vaccines, those derived from PPRV/Nigeria/75/1 (N75) and PPRV/India/Sungri/96 (S96), elicited both PPRV‐specific antibody and CD8^+^ T‐cell responses, while S96 also elicited a significant PPRV‐specific CD4^+^ T‐cell response (Hodgson et al., 2018). The protection from challenge provided by the vaccines may be due to any or all of these immune responses. Determination of these immune correlates of protection is important in the case of the PPR control programme as the current vaccines do not allow the differentiation of previously infected animals from those that have been vaccinated, the so‐called DIVA (Differentiation of Infected and Vaccinated Animals) capability. This capability will be in demand during the closing stages of the PPR eradication campaign, and after eradication, when use of live PPRV vaccines is likely to be banned (Baron et al., 2017). Work is therefore ongoing in several laboratories to create a new anti‐PPRV vaccine that can function as a DIVA vaccine (Caufour et al., 2014; Diallo et al., 2002; Fakri et al., 2018; Herbert et al., 2014; Qin et al., 2012; Rojas et al., 2014; Wang et al., 2013), and the requirement to test new vaccines by exposing animals to wild‐type PPRV, a procedure requiring high containment facilities in most countries, could be minimized if it was possible to demonstrate that the new vaccine elicited an immune response known to be protective.

For studies in mice or other in‐bred animals, information on the relative contribution to protection made by antibodies, CD4^+^ T cells and CD8^+^ T cells has been obtained by passive/adoptive transfer of specific components from vaccinated animals to naive. In the case of outbred animals such as goats, sheep and cattle, adoptive transfer of immune cells is not possible, or at the very least problematic, due to the immune response of the recipient animals to alloantigens on cells from the (genetically distinct) donors. An alternative approach has been to use the infusion of complement‐fixing monoclonal antibodies (mAbs) to cause the lysis of specific T‐cell subsets to deplete them from the experimental animal to see whether they play a role in protection from disease in either naive or vaccinated animals (Naessens et al., 1998). Examples of such studies are the finding that removal of CD4^+^ T cells from sheep and goats immunized against the nematode parasite *Haemonchus contortus* partially abrogated protection (Karanu et al., 1997) and that depletion of CD8^+^ T cells from macaques vaccinated with a recombinant adenovirus expressing Ebola virus glycoprotein GP abrogated protection against Ebola virus (Sullivan et al., 2011).

We show here that depletion of CD8^+^ T cells from S96‐vaccinated goats has no effect on protection from challenge with wild‐type PPRV, indicating that the primary protection is provided by antibody and/or CD4^+^ T cells.

## 2 MATERIALS AND METHODS

### 2.1 Viruses, including vaccines

All viruses used in this study were as previously described (Hodgson et al., 2018).

### 2.2 Monoclonal antibodies

Two mAbs were used in CD8 T‐cell depletion studies: (a) CC63, which recognizes the alpha chain of bovine CD8 (MacHugh et al., 1991) and cross‐reacts with caprine CD8 (Davis & Ellis, 1991); (b) TRT3 (Cook et al., 1993), which recognizes a turkey rhinotracheitis virus protein and was used as an isotype‐matched control antibody. CC63 for the pilot study was purified in‐house: hybridoma culture supernatant from clone CC63 (IgG2a) was prepared in the institute's Monoclonal Antibody Unit and IgG purified by chromatography on a 5 ml Protein G column (GE Healthcare) according to the manufacturer's instructions. The eluted IgG was dialysed overnight against PBS and the protein content determined using a Coomassie (Bradford) Protein Assay kit (Thermo Fisher). The larger stocks of CC63 and TRT3 required for the main experiment were prepared commercially under contract with Antibody Production Services Ltd.

For labelling different leucocyte subsets in whole blood, the mAbs used were as follows: anti‐ovine CD4 clone 17D (Mackay et al., 1988) (cell line ECACC 91060551 obtained from European Collection of Authenticated Cell Cultures); anti‐ovine WC1 clone 197 (McClure & Hein, 1989); anti‐ovine MHC class II (MHCII) clone H42A (Washington State Monoclonal Antibody Center); anti‐bovine CD8 clone CC58 (MacHugh & Sopp, 1991); and anti‐bovine CD21 clone CC21 (Naessens et al., 1990). CC58 recognizes the beta chain of CD8 (MacHugh et al., 1991) and is not competed by CC63 (Hope et al., 2002; Juleff et al., 2009). CC58 and CC21 cross‐react with caprine CD8 and CD21, respectively (Davis & Ellis, 1991).

Mouse mAbs were conjugated to Alexa Fluor 488 (AF488) using a specific antibody labelling kit (Thermo Fisher), while mAb conjugation with other fluorochromes (allophycocyanin (APC), R‐phyco‐erythrin (RPE)) was carried out using Lightning Link kits (Innova Biosciences); in all cases, the manufacturers' instructions were followed directly. Antibodies and techniques for intracellular labelling of gamma interferon (IFN‐γ) were as previously described (Hodgson et al., 2018).

### 2.3 Animal studies

All animal studies were carried out under licences PPL 70/7199 and PPL70/8833 issued by the Home Office of the United Kingdom in accordance with relevant legislation and after approval by the Pirbright Institute Animal Welfare and Ethical Review Board. Outbred goats were sourced from commercial farms. Animals entered the secure isolation units 7 days before the start of the procedure to acclimatize to the new husbandry regime and were provided with daily enrichment during the trials. All experimental goats were male outbred UK whites, a Saanen‐derived cross, and were 9–12 months old at the start of the study.

For depletion studies, all mAb preparations were given by intravenous inoculation. All animals were observed for at least 30 min after inoculation of mAb.

#### 2.3.1 Pilot study

Two different protocols were assessed using two animals each. On day 1 (D1), all four goats received two injections of CC63, 0.5 mg in the morning and 1.5 mg in the afternoon. Two animals were given further high doses of mAb (25, 20, 20 mg) on D2–D4 (high‐dose protocol), and two animals were given 2 mg per day each day on D2–D10 (low‐dose protocol). All animals received Flunixin (Finadyne™) at 2.2 mg/kg prior to mAb inoculation for the first 3 days to alleviate the inflammatory effects of cell lysis induced by the inoculated mAb. Blood was taken into EDTA vacutainers on D0–D4, D8 and D10 to assess the efficiency of CD8^+^ T‐cell depletion. One animal from each group was euthanized on D5 and the remaining animals at D10.

#### 2.3.2 Full scale study of effect of CD8^+^ T‐cell depletion

Ten goats were vaccinated with 2 × 10^4^ TCID_50_ S96 in a 1 ml volume inoculated subcutaneously; one of these animals proved impossible to get regular blood samples from, due to the depth of its veins and its temperament, and was removed from the study. Two further goats were not vaccinated and acted as challenge controls. Whole blood for serum and heparinized blood for purification of peripheral blood mononuclear cells (PBMCs) were collected weekly from the jugular vein.

Twenty six days after vaccination (2 days before challenge), the vaccinated goats were given 2 mg of either CC63 (*n* = 5) or TRT3 (*n* = 4), in each case as two doses, 0.5 and 1.5 mg, as on D1 in the pilot study. The vaccinated goats were further inoculated with their respective mAbs on the three subsequent days, receiving two successive doses of 20 mg, and a final dose calculated so that the total amount of mAb for each animal was of 2.1 mg mAb/kg body weight. All animals received Flunixin (Finadyne™) at 2.2 mg/kg prior to each inoculation of mAb; non‐vaccinated controls, which did not receive mAb, were also treated with Flunixin at the same time in case this treatment should have any effect on clinical signs due to PPRV challenge. Twenty eight days after vaccination, all goats were infected with the virulent field strain PPRV/Sudan/Sennar/72 (2 × 10^5^ TCID_50_ in 1 ml) by the subcutaneous route. Blood was collected into EDTA vacutainers at −5, −2, −1, 0, 2, 4, 6, 8, 10 and 12 days post‐challenge (dpc), equivalent to 23, 26, 27, 28, 30, 32, 34, 36, 38 and 40 days post‐vaccination (dpv). Clinical assessments were carried out daily as previously described (Hodgson et al., 2018). All animals were euthanized at 42 dpv by overdose of pentobarbital.

### 2.4 Whole blood cell surface membrane protein labelling

For the studies of leucopenia following infection with wild‐type PPRV, triplicate samples (100 μl) of EDTA blood were mixed with 20 μl of either mAb cocktail A (APC‐labelled anti‐CD4 mAb, AF488‐labelled anti‐CD8 mAb and RPE‐labelled anti‐CD21 mAb) or mAb cocktail B (APC‐labelled anti‐WC1 mAb and AF488‐labelled anti‐MHCII mAb). For the studies on CD8^+^ T‐cell depletion, a single mAb cocktail containing APC‐labelled anti‐CD4, AF488‐labelled anti‐CD8 and RPE‐labelled anti‐WC1 was used. The blood samples were gently mixed with antibody in polystyrene flow tubes (BD Biosciences) and incubated at room temperature in the dark for 30 min. Two ml of Fix/Lyse buffer (eBioscience) was added to the blood/antibody mixes to lyse the red blood cells and fix the samples for analysis. Tubes were incubated for a further 30 min at room temperature in the dark, after which 100 μl 123count eBeads™ (eBioscience) were added to the tubes. Flow cytometry data were collected using an LSRFortessa (BD Biosciences) using FACSDiva software (v8), with 10,000 events being obtained in the 123count eBead gate. Flow cytometry files were analysed in FlowJo X. A representative data set showing the gating strategy is shown in Figure S1. Numbers of the different cell populations present within whole blood samples were calculated according to the formula: number of cells per ml of blood = (cell count/eBead count) × eBead concentration.

### 2.5 Other assays

Preparation of viral antigen, ELISA for anti‐PPRV antibodies, assay of T‐cell proliferation and real‐time PCR for PPRV genome are all as previously described (Hodgson et al., 2018).

### 2.6 Statistical analyses

Statistical analyses of data from animal studies were carried out essentially as previously described (Hodgson et al., 2018). For statistical analysis of the study of PPRV‐induced leucopenia, differences between the response over time of vaccinated and unvaccinated animals were assessed by ANOVA comparison of linear models with or without a component for interaction of time and vaccination status; all the data were considered as coming from a single study, and all *p* values given in the text are those after adjustment for multiple tests using the method of Holm (Holm, 1979), as implemented in the R function *p.adjust*. For statistical analysis of the changes in the concentration of CD8^+^, CD4^+^ and WC1^+^ T cells in whole blood during the depletion and challenge, the data were fit to linear mixed models in which depletion group and dpv were fixed factors and experimental animal was a random factor and the models used to examine the statistical significance of differences between the two depletion groups and changes to the cell count over time. All the comparisons were considered as belonging to a single group, and the p values given in the text are again the values after adjusting for multiple comparisons using the method of Holm.

## 3 RESULTS

### 3.1 Effect of wild‐type PPRV infection on different leucocyte subsets

**FIGURE 1:**
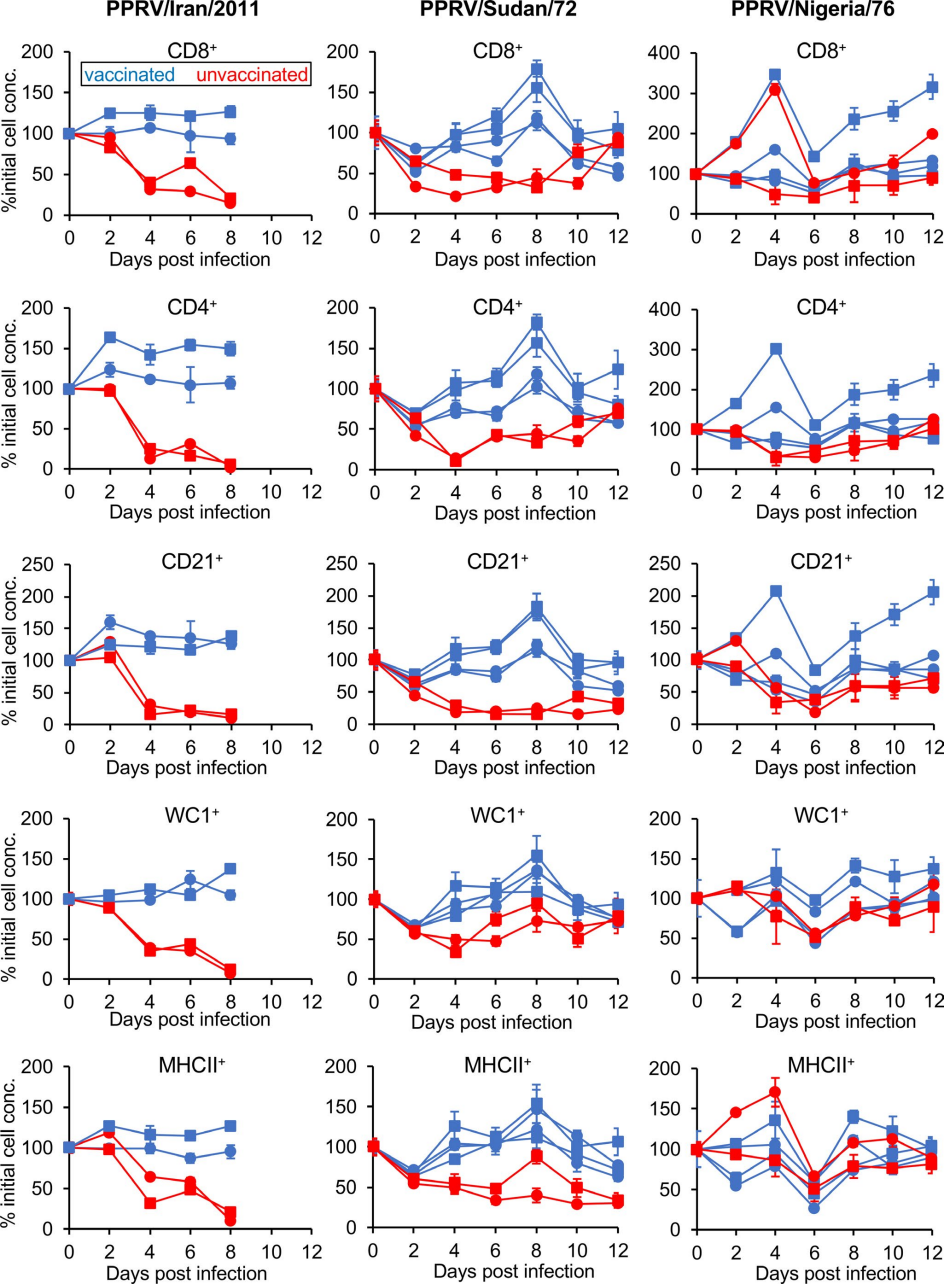
Levels of different leucocyte subsets during PPRV infection of goats. The concentrations of 5 different leucocyte subsets (CD8^+^ and CD4^+^ T cells, CD21^+^ B cells, WC1^+^ γ/δ T cells and MHCII^+^ antigen‐presenting cells) in whole blood were measured, as described in Materials and Methods, in groups of animals infected with one of three strains of PPRV: Iran/2011, Sudan/72 or Nigeria/76. The changes occurring in naive infected animals were compared with those in animals that had previously been vaccinated against wild‐type PPRV using attenuated PPRV vaccines. To allow for the wide variation in normal cell concentrations between animals, the concentrations were normalized between animals by setting to % of the pre‐infection concentration (% initial cell conc.)

Peste des petits ruminants virus, as with all the morbilliviruses, causes a rapid loss of circulating white blood cells during infection (see, e.g. Baron et al., 2014). There have been no studies of which leucocyte subtypes are most affected during this leucopenia, or whether any cell subset is affected even in vaccinated animals, in which, normally, little or no leucopenia is observed. In order to establish a baseline of what is happening to circulating leucocytes during PPRV infection, we used samples taken from both vaccinated and unvaccinated animals during three of the challenge trials carried out for our previous study (Hodgson et al., 2018) and quantified the numbers of cells carrying specific cell surface protein markers per ml of whole blood. The cell subsets studied were limited by the availability of appropriate antibodies reacting with goat leucocyte markers, but we were able to quantify CD4^+^ and CD8^+^ T cells, WC1^+^ cells (a major subset of γδ T cells), CD21^+^ B cells and MHCII^+^ cells (this last as a marker for antigen‐presenting cells). We found that the PPRV‐induced leucopenia following infection affected all of these leucocyte subsets, although the extent of the effect was dependent on the pathogenicity of the virus (Figure 1). For animals infected with either PPRV/Iran/2011 or PPRV/Sudan/72, there was a clear and statistically significant difference between the time profiles of the vaccinated and non‐vaccinated animals for all five cell types (*p* < .001 in all cases) (Figure 1). The highly pathogenic PPRV/Iran/2011 strain caused an almost complete loss of all five leucocyte subsets by 8 dpc, at which time the disease had become so severe that the animals had to be euthanized. The PPRV/Sudan/72 strain, which causes a moderate disease, also led to a reduction in numbers of all five cell subsets, though the loss of specific cell types was less pronounced in this case. The numbers of CD4^+^, CD8^+^ and WC1^+^ cells can be seen to be recovering by 12 dpc, although the numbers of CD21^+^ and MHCII^+^ cells did not recover over this time period. In contrast, in animals infected with the very mild Nigeria/76/1 strain, the leucopenia was much less marked. There was no significant difference between vaccinated and non‐vaccinated animals for CD8^+^ or WC1^+^ T cells, or MHCII^+^ antigen‐presenting cells, and the differences for CD4^+^ cells and CD21^+^ cells were small, even if statistically significant (*p* = .0005 and *p* = .013, respectively).

### 3.2 Pilot study of CD8^+^ T‐cell depletion in goats

While mAb CC63 has previously been used successfully to deplete CD8^+^ T cells in cattle (Juleff et al., 2009; Taylor et al., 1995; Thomas et al., 1996) and has been shown to cross‐react with caprine CD8 (Davis & Ellis, 1991), it had not been formally shown to lead to depletion of CD8^+^ T cells in small ruminants. In addition, two different kinds of protocol have been used successfully to deplete CD8^+^ T cells in cattle and sheep, a short high‐dose protocol, where large amounts of antibody are administered over 1–4 days (e.g. Carr et al., 2013; Juleff et al., 2009; Naessens et al., 1998; Sacchini et al., 2011), or a longer, low‐dose protocol, where smaller amounts of mAb are administered every day over a longer period (e.g. Eriksson et al., 1999; Taylor et al., 1995; Thomas et al., 1996). We therefore conducted a pilot study to determine which protocol was most effective when using CC63 in goats. Two animals were treated with each protocol as described in Materials and Methods. Samples of blood were taken before administration of antibody and at 1, 2, 3, 4, 8 and 10 days after the first injection of CC63 to quantitate the levels of CD8^+^ T cells. CD4^+^ T cells and WC1^+^ (γδ) T cells were also quantified for comparison.

**FIGURE 2:**
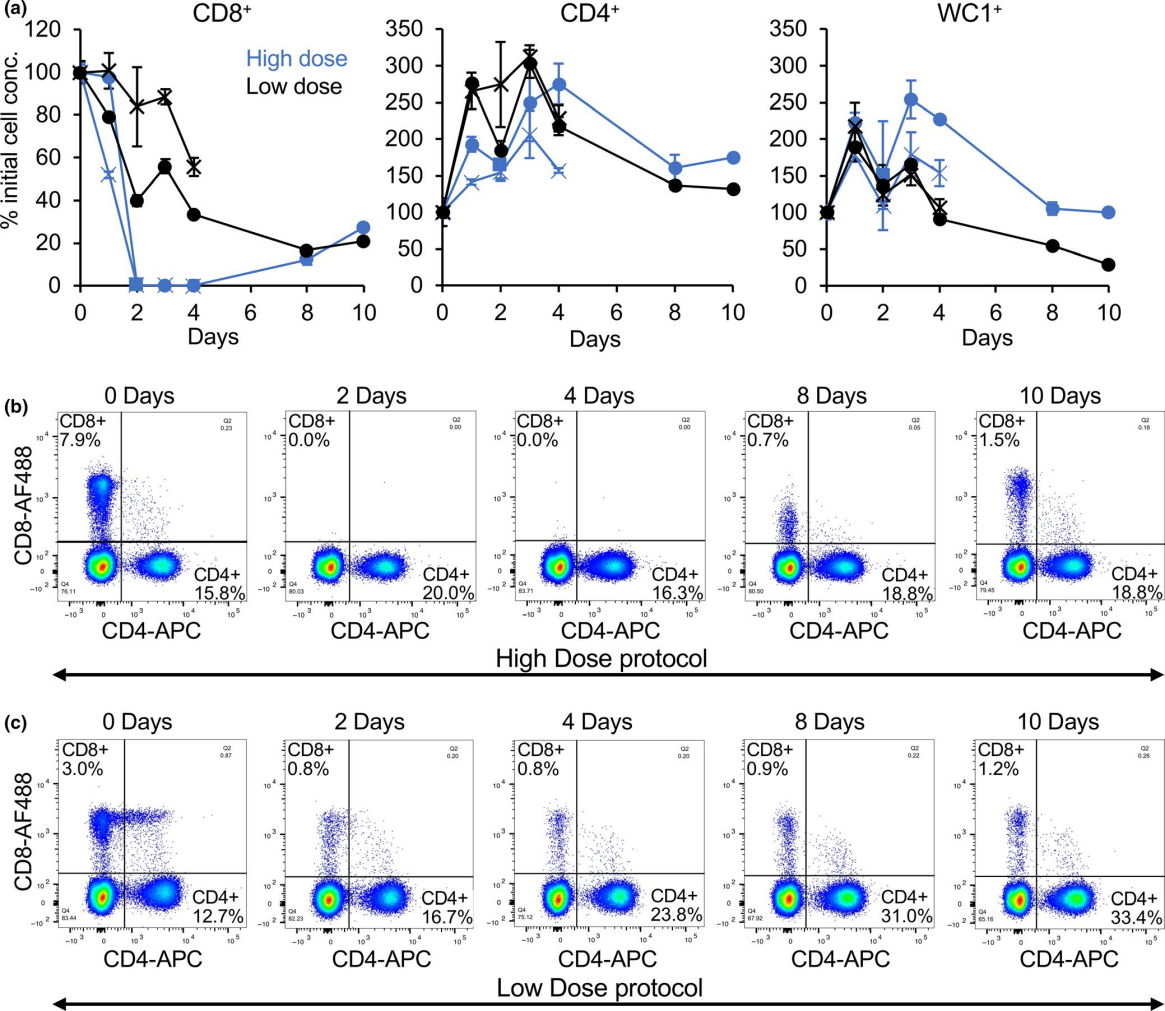
Pilot study of depletion of CD8^+^ T cells in goats. Goats were treated with two different protocols to deplete CD8^+^ T cells, as described in Materials and Methods. (a) At the indicated times, the concentration of CD8^+^, CD4^+^ and WC1^+^ T cells in whole blood was determined and shown in blue for the animals given the high‐dose protocol and in black for animals given the low‐dose protocol; to allow for the wide variation in normal cell concentrations between animals, the concentrations were normalized between animals by setting to % of the pre‐treatment concentration (% initial cell conc.). (b, c) Examples of flow cytometry data from animals given the high‐dose (b) or low‐dose (c) protocols, illustrating the relative loss of CD8^+^ T cells in the two protocols

Observation of the experimental animals showed that, even though they had been pre‐treated with a strong anti‐inflammatory drug, all animals showed signs of transient physiological changes such as increased respiratory rate and abdominal breathing after each of the first few injections of CC63, signs commensurate with large scale cell lysis. The concentrations of the three cell types at each day are shown in Figure 2a; because of the very variable initial concentration of white cells in different experimental animals, these counts have been normalized as % of the initial (day 0) count to allow clearer display. Figure 2b,c shows representative examples of the actual cell labelling data for CD8^+^ and CD4^+^ cells from animals given the high‐ and low‐dose protocols, respectively. After 2 days of treatment, numbers of CD8^+^ T cells in both animals on the high‐dose protocol had been reduced to undetectable levels (Figure 2a,b), where they remained until at least day 4. In the low‐dose group; however, levels of CD8^+^ T cells fell more slowly, still being clearly detectable at day 4. One animal in each group was sacrificed at day 5 for post‐mortem examination. In the remaining animal given the high‐dose protocol, the numbers of CD8^+^ T cells had begun to increase at day 8 and were clearly recovering at day 10. In the animal being treated with the low‐dose protocol, the levels of the CD8^+^ cells were never reduced below 20% of their initial level and were also beginning to increase at day 10.

In contrast to the loss of CD8^+^ T cells, the numbers of CD4^+^ T cells increased nearly twofold immediately after the first injections of mAb in all treated animals; while levels of these cells began to fall after 4 days, they had not returned to baseline at the end of the study. Levels of WC1^+^ cells were not significantly affected at the beginning of the study, but were seen to be reduced at day 10 in both surviving animals (Figure 2).

Based on this study, the short high‐dose protocol was considered the more effective at depleting the goat CD8^+^ T cells and would provide a window of at least 4 days when essentially all CD8^+^ T cells would be removed, a window larger than that required for PPRV infection to become established. This protocol was therefore adopted for the main experiment.

### 3.3 Depletion of CD8^+^ T cells in vaccinated goats and wild‐type PPRV challenge

To assess the role of CD8^+^ T cells in vaccine‐induced protection, nine goats were vaccinated with the standard dose of live attenuated PPRV/India/Sungri/96 vaccine. These animals were randomly allocated to one of two groups, CD8 depletion (*n* = 5) and mock‐depletion (*n* = 4). Two additional animals were mock vaccinated with a control preparation made from the cells used to grow the vaccine virus; these animals were used to confirm the validity of the wild‐type virus challenge. From 26 to 29 dpv (−2 to 1 dpc), the CD8‐depletion group was treated with CC63; the final dose of antibody for each animal was adjusted so that each received 2.1 mg/kg body weight, as antibody doses >2 mg/kg have previously been shown to deplete CD8^+^ T cells not only from the circulating blood but also from the lymphatic organs (Naessens et al., 1998). At the same time, the mock‐depletion group was treated with an identical regimen of inoculations using an isotype control mAb (TRT3, which recognizes a protein not found in either PPRV or goats).

**FIGURE 3:**
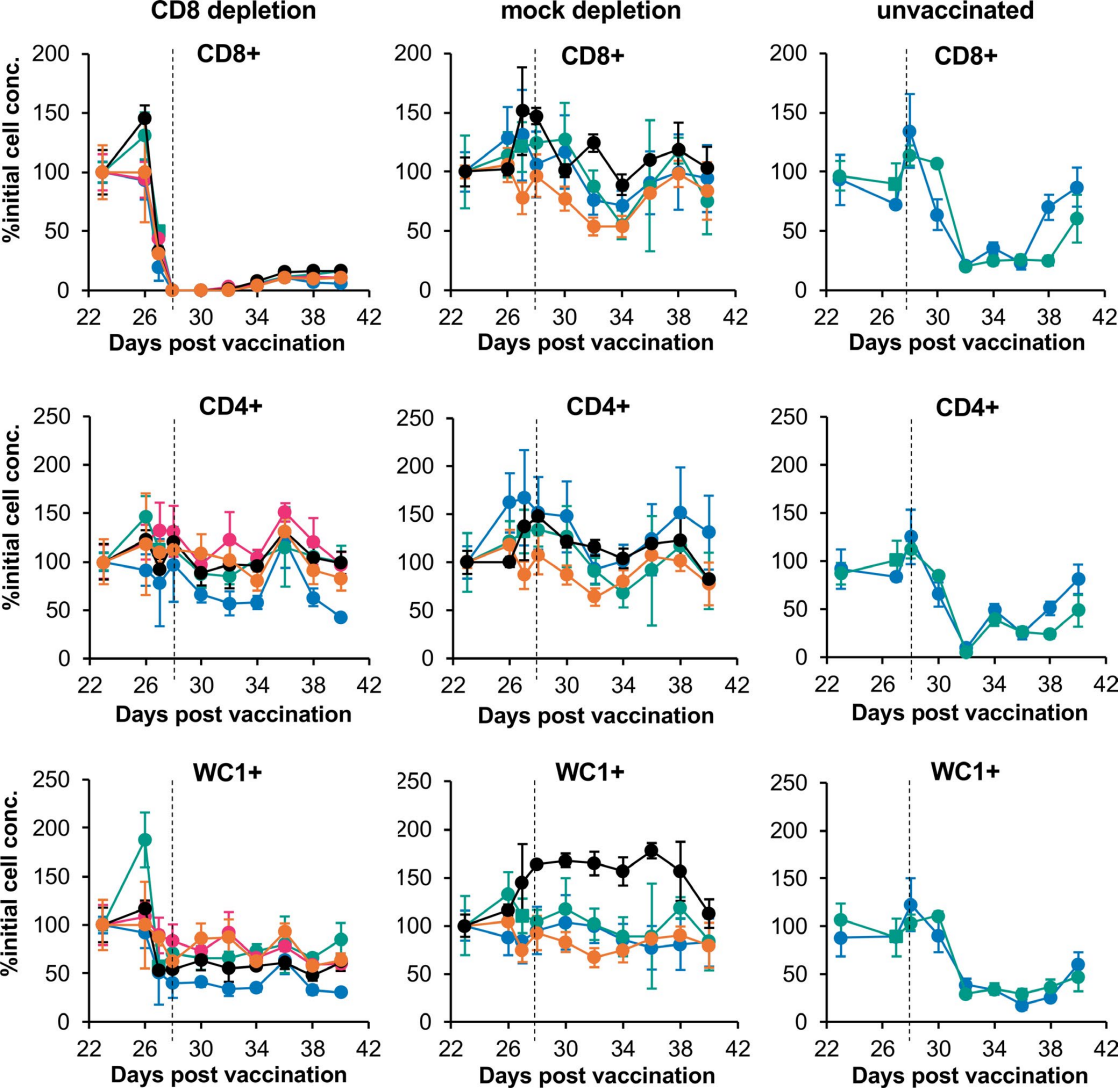
T‐cell numbers over course of depletion‐challenge. Vaccinated goats were treated as described in Materials and Methods to deplete CD8^+^ T cells (CD8 depletion) or given equal doses of a control antibody (mock depletion). Levels of CD8^+^, CD4^+^ and WC1^+^ T cells were determined before (23 dpv) and after the start of the depletion protocol at 26 dpv. The concentrations of these cell types were also measured in two unvaccinated animals that were used as infection controls. To allow for the wide variation between animals of the normal cell concentrations, the cell concentrations were normalized between animals by setting to % of the pre‐treatment concentration (% initial cell conc.). At 28 dpv (vertical dotted line on graphs), all the animals were infected with PPRV/Sudan/72

At 28 dpv, all animals were infected with PPRV/Sudan/Sennar/72, a virus known to have intermediate virulence in these goats (Baron et al., 2014; Hodgson et al., 2018). As expected from our earlier studies (Figure 1), the two unvaccinated infection control animals showed transient loss of all three cell types being measured (Figure 3), starting at 4 dpc (32 dpv) and recovering to >50% of normal levels by 14 dpc (42 dpv).

For the CD8 depletion group, a decrease in CD8^+^ T‐cell numbers compared to baseline was already apparent in all animals at 27 dpv, 1 day after the initial inoculation of 2 mg of mAb (Figure 3). On the day of challenge, CD8^+^ T cells in the blood of goats in this group had been reduced from an average of 7 × 10^6^ cells/ml whole blood to an average of 6.6 × 10^3^ cells/ml, a reduction of 99.91%. CD8^+^ T cells remained profoundly depleted in the peripheral blood of all goats of this group until at least 32 dpv (4 dpc), returning to an average of 5.4 × 10^5^ cells/ml (<10% baseline) in these animals at 38 and 40 dpv. The reduction in CD8 count relative to the pre‐depletion level was significant at all times from 27 dpv onward (*p* < 10^–17^ at each time point). Relative to the pre‐treatment count, there was no significant change in the numbers of CD8^+^ T cells in the blood of the mock‐depletion group at any time point apart from a transient decrease at 34 dpv (*p* = .022).

No change was seen in the concentration of CD4^+^ T cells over the course of the treatment with mAb and challenge with wild‐type PPRV in either depletion group. There was no difference between the two groups, and there was no statistically significant change in the numbers of CD4^+^ T cells on any day relative to the pre‐treatment levels. It is not clear why we did not observe, in the CD8 depletion group, the rise in CD4^+^ cells seen in the pilot experiment, but it may be due to the more intensive efforts we made in this study to control the inflammatory response caused by the lysis of CD8^+^ cells. In contrast to the CD4^+^ cells, the WC1^+^ T‐cell count in the CD8‐depletion group dropped ~40% at 27 dpv and then remained at that level for the rest of the study, while the mock‐depletion group showed no change in the count of WC1^+^ T cells over the period of the study. This change was statistically significant for all days from 27 dpv on (*p* < 10^–8^ at all times except 36 dpv, when *p* = 10^–4^) and was consistent in all five animals. It appeared to be associated with the initial loss of CD8^+^ T cells, as it clearly preceded the challenge infection at 28 dpv (Figure 3).

**FIGURE 4:**
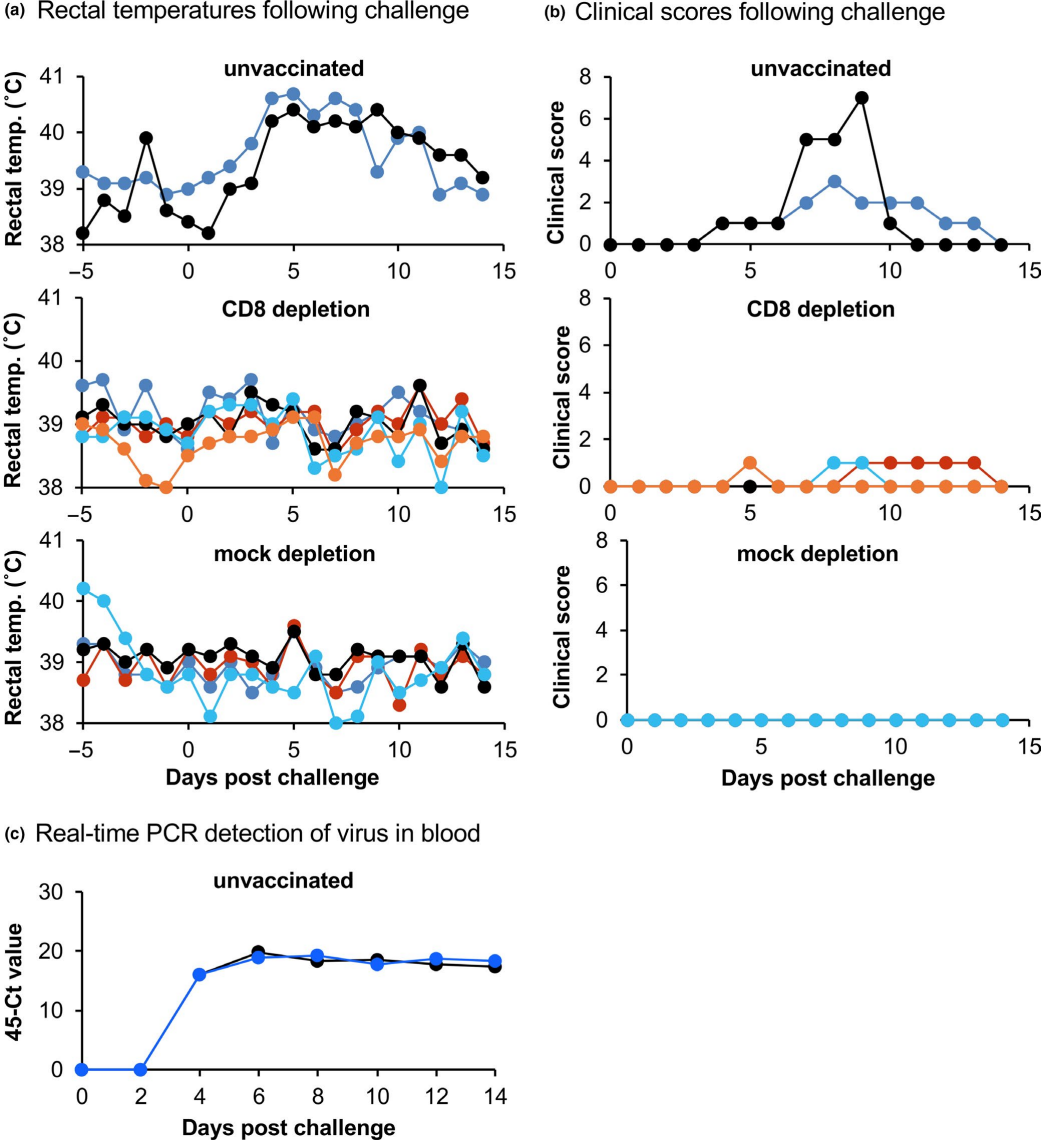
Clinical signs and viraemia during challenge. For the goats treated as described in the legend to Figure 3, the rectal temperatures (a) and clinical scores (b) were determined after challenge infection with PPRV/Sudan/72. (c) Levels of PPRV genome in the blood of infected animals were determined by RT‐qPCR. Samples from the vaccinated animals (both CD8 depletion and mock‐depletion groups) were negative for PPRV RNA at all times (not shown)

All 11 goats were observed daily for clinical signs. The first such signs were observed in the unvaccinated controls at 4 dpc, when the rectal temperatures of both goats increased by more than 1.5°C above baseline; they remained above baseline for the remainder of the study (Figure 4). At 7 dpc, these animals developed nasal congestion and subsequently also diarrhoea. The mock‐depletion goats did not display any outward clinical signs during the challenge observation period and their rectal temperatures did not increase above baseline observations (Figure 4). Three out of five of the goats in the CD8‐depletion group developed loose faeces for at least 1 day during the 2 week observation period, but they did not develop nasal or ocular congestion, nor did their rectal temperatures increase (Figure 4). Diagnostic real‐time PCR for the detection of PPRV genome RNA was positive for the two unvaccinated controls from 4 dpc to 14 dpc (Figure 4c), but no PPRV RNA was detected in the blood taken from either the CD8 depletion or mock‐depletion groups at any time point (data not shown).

### 3.4 Immune responses following vaccination and challenge

**FIGURE 5:**
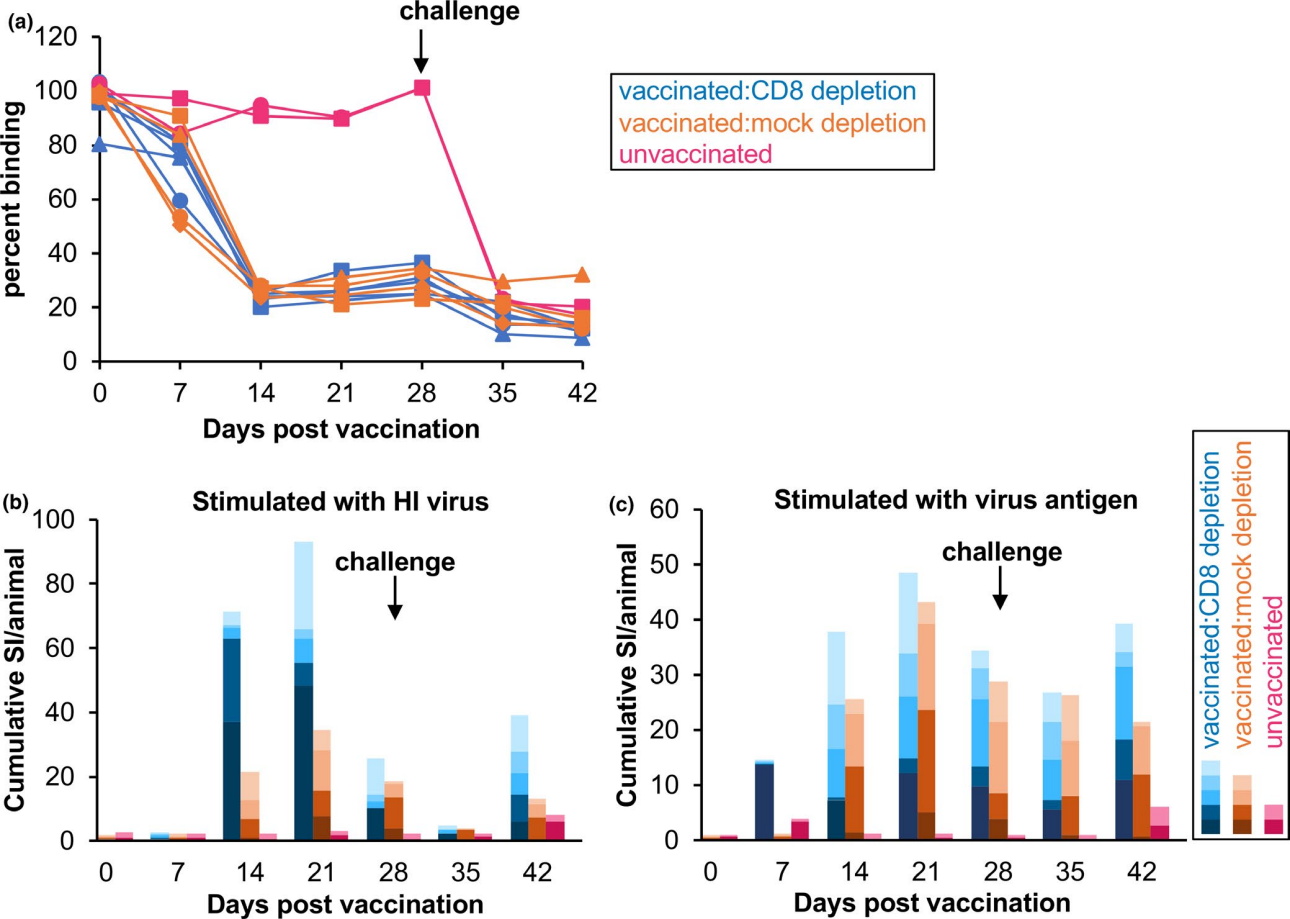
Antibody responses and proliferation responses in vaccinated and unvaccinated goats. The PPRV‐specific immune responses were measured for all animals in terms of (a) the PPRV‐specific antibody response and (b, c) the PPRV‐specific T‐cell response. Depletion of CD8^+^ cells, or mock depletion, occurred before challenge, which was at 28 dpv (arrow). (a) Levels of anti‐PPRV antibody were determined for all animals using competition ELISA (cELISA); for this assay, a reduction in binding of the specific monoclonal antibody indicates the presence of competing anti‐PPRV antibodies. (b, c) Proliferation of purified leucocytes in response to stimulation with (b) heat‐inactivated (HI) virus or (c) an inactivated preparation of virus protein (virus antigen). Proliferation is shown as the cumulative stimulation index (SI) for each group of animals, normalized to the number of animals in each group

The vaccinated animals, as seen in our previous studies (Hodgson et al., 2018; Holzer et al., 2016), developed a strong anti‐PPRV antibody response as measured by cELISA specific for antibodies to the PPRV N protein (Figure 5a). Both groups of vaccinated animals showed a further increase in anti‐PPRV antibodies after challenge, and there was no significant difference between the antibody responses in the CD8^+^ T‐cell‐depleted group and the mock‐depleted group. The unvaccinated animals showed no anti‐PPRV antibody until after challenge, but responded strongly to the challenge, having levels of anti‐PPRV antibody at 7 dpc that were the same as those seen in previously vaccinated animals.

The vaccinated animals also showed a clear T‐cell proliferative response to both PPRV antigen (protein from PPRV‐extracted cells) and heat‐inactivated (HI) virus, reaching maximum levels at around 21 dpv (Figure 5b,c). As observed previously (Hodgson et al., 2018), the response to crude virus antigen was in general more robust than that to heat‐inactivated virus, apart from one animal which showed a very strong response to HI virus at 14 and 21 dpv. No significant increase in the proliferative response was seen after challenge, and there was no effect of CD8^+^ T‐cell depletion (no significant difference between CD8‐depleted and mock‐depleted groups at 28, 35 or 42 dpv), confirming our previous findings that the major component of this proliferation response is most likely to be CD4^+^ T cells. The unvaccinated controls showed no response in this assay, the small apparent increase in the stimulation index (SI) seen at 42 dpv (14 dpc) for samples from these two animals not being statistically significant.

**FIGURE 6:**
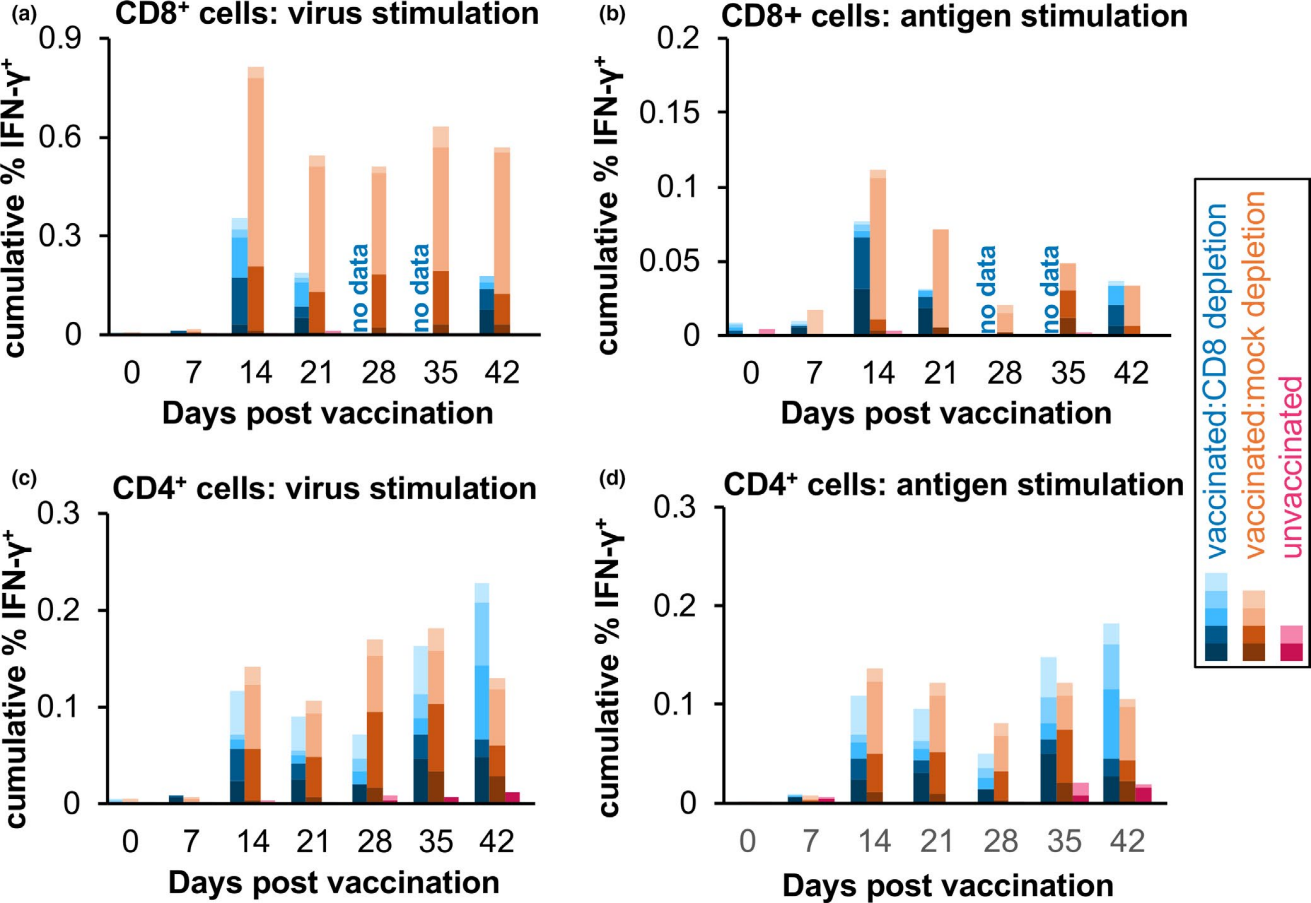
IFN‐γ responses in CD4^+^ and CD8^+^ T cells in vaccinated animals. The fraction of (a, b) CD8^+^ and (c, d) CD4^+^ T cells that were IFN‐γ^+^ after stimulation of PBMCs with (a, c) live PPRV (virus) or (b, d) inactive virus antigen (antigen) was measured by labelling with immunofluorescent mAbs and flow cytometry. The % IFN‐γ^+^ cells in each case was calculated as the average of the specific response to N75 or S96 virus/antigen, where specific response is (response in the presence of virus/antigen) – (response in the presence of control preparation). The cumulative per cent of the parent cell type that was positive for IFN‐γ is shown for each group of animals, normalized by the number of animals in the group

PPRV‐specific CD4^+^ and CD8^+^ T cells could be detected in vaccinated animals by intracellular labelling of IFN‐γ in antigen‐stimulated PBMC (Figure 6). As previously seen (Hodgson et al., 2018), live virus was better than inactivated virus antigen at activating IFN‐γ secretion in CD8^+^ T cells (Figure 6a). Although one animal in the mock‐depletion group showed a particularly strong PPRV‐specific CD8^+^ T‐cell response in this assay, there was no statistically significant difference in the numbers of PPRV‐specific CD8^+^ or CD4^+^ T cells in the two groups of vaccinated animals at any day post‐vaccination and pre‐depletion. Both groups of animals showed significant numbers of PPRV‐stimulated CD8^+^ and CD4^+^ T cells at 14 and 21 dpv.

As expected from the data in Figure 3, the number of CD8^+^ T cells detected by flow cytometry in the PBMCs from the animals in the CD8 depletion group was very low from 28 dpv onwards (Figures S2 and S3), even though preparation of PBMCs provides a partial concentration of T cells compared with whole blood. Given that the numbers of IFN‐γ^+^CD8^+^ T cells in the mock‐depletion animals were only 40–50 per sample (Figure S2), this meant that the median number of such cells seen at 28 and 35 dpv in the CD8 depletion group was less than two. The fraction of CD8^+^ T cells in these samples responding to PPRV could therefore not be estimated, and this is shown as ‘no data’ in Figure 6a,b. By 42 dpv, the numbers of CD8^+^ T cells in the CD8 depletion group were beginning to recover (Figure 3), and we included the data for these cells in Figure 6, although the number of events was still very small (Figure S2). Nevertheless, it is clear from Figure 6 that the vaccination had elicited both PPRV‐specific CD4^+^ T cells and PPRV‐specific CD8^+^ T cells, as seen in our previous studies (Hodgson et al., 2018), and therefore that the depletion of CD8^+^ T cells had largely eliminated a part of the vaccine‐induced immune response.

Neither group of vaccinated animals showed a significant increase in the virus‐specific CD4^+^ T‐cell response after challenge, nor was there any significant increase in the numbers of PPRV‐specific CD8^+^ T cells after challenge. The unvaccinated animals did not have significant numbers of PPRV‐specific IFN‐γ^+^ T cells of either type on any day.

## 4 DISCUSSION

There have been a number of studies on the effects of depleting CD4^+^ or CD8^+^ T cells from different types of livestock animals, but most of these have been aimed at determining the role played by these cells in protection from primary infection and have therefore depleted the cells from naive animals. In this way, it has been shown, for example, that depletion of CD8^+^ T cells from cattle had no effect on trypanosome infections (Sileghem & Naessens, 1995), but delayed clearance of bovine respiratory syncytial virus infections (Taylor et al., 1995). Depletion of CD4^+^ T cells from cattle had little effect on the control of primary infections with *Mycoplasma mycoides* subsp. *mycoides*, which causes contagious bovine pleuropneumonia (Sacchini et al., 2011), while depleting these cells from sheep made them more susceptible to the intestinal parasite *H, contortus* (Pena et al., 2006). Similar studies, in which depletion is carried out at the time of vaccination, have been carried out to determine whether specific types of T cell are required for the vaccine to elicit a protective immune response (e.g. Carr et al., 2013; Elong Ngono et al., 2019; Valkenburg et al., 2018).

A few studies have focussed on vaccinated animals, and the results from these have generally confirmed the understanding that eliciting pathogen‐specific CD8^+^ T cells requires active synthesis of suitable proteins in the hosts’ cells. In addition to those studies mentioned in the Introduction, CD8^+^ T cells were shown to be required for full protection against *Yersinia pestis* in mice that had been vaccinated using a DNA construct (Wang et al., 2011), while depletion of CD4^+^ T cells abrogated protection against *Aspergillus fumigatus* (which causes the internal fungal infection aspergillosis) in mice that had been given a subunit vaccine (Diaz‐Arevalo et al., 2011). In cases where a live virus vaccine has been used, both cell types may play a role, as in orthopoxvirus infection, where depletion of either CD8^+^ or CD4^+^ T cells affected protection from secondary infection (Chaudhri et al., 2015).

The only vaccines against PPRV that are in field use are live attenuated strains of the virus. In this respect, PPRV is similar to other morbilliviruses, where live attenuated strains of MV, CDV and (before it was eradicated) RPV are the main methods of controlling the respective diseases. Various vectored vaccines, based on recombinant pox viruses or adenoviruses encoding morbillivirus glycoproteins, have been shown to be effective against the respective morbillivirus (e.g. Caufour et al., 2014; Fischer et al., 2002; Fooks et al., 1998; Herbert et al., 2014; Qin et al., 2012; Taylor et al., 1991), but have never been put to use in the field. In the case of PPRV, the rules for vaccines are set out in the Terrestrial Manual published annually by the World Organisation for Animal Health, where an effective (live attenuated virus) vaccine is required to elicit a minimum titre of neutralizing antibodies. This is sometimes interpreted to mean that neutralizing antibodies are what is required for protection, whereas all it actually indicates is an easily‐measured sign that the vaccine has been successfully administered. The attenuated PPRV vaccines are known to elicit specific antibodies as well as PPRV‐specific CD4^+^ and CD8^+^ T cells (Hodgson et al., 2018). It is not yet established whether neutralizing antibodies are critical for protection against morbilliviruses: vaccination with baculovirus‐expressed RPV glycoproteins elicited neutralizing antibodies in cattle but did not protect against infection (Bassiri et al., 1993), while in humans it is well‐established that the ability to make antibody is not required for clearing MV in normal infections or for subsequent protection (Good & Zak, 1956), and it has been shown that neutralizing antibodies are neither necessary nor sufficient to protect against MV challenge in a rodent model (Brinckmann et al., 1991). On the other hand, studies in a macaque model of MV infection showed that vaccine that elicited MV‐specific CD4^+^ and CD8^+^ T cells, but not neutralizing antibody, failed to protect against subsequent challenge (Lin et al., 2014). Protection provided by the attenuated PPRV vaccines may therefore be mediated at least in part by the antibody that it elicits, but may also depend on either or both of the types of specific T cells produced.

We have shown that elimination of CD8^+^ T cells from animals vaccinated with a live attenuated PPRV vaccine had only a slight effect, if any, on protection from subsequent challenge with a pathogenic virus. Goats that had been vaccinated and then depleted of 99.9% of their CD8^+^ T cells before challenge showed no sign of viraemia, no pyrexia and no major clinical signs, apart from a very mild and transient diarrhoea in three out of five animals, which may point to a particular role of previously primed CD8^+^ T cells in clearing PPRV from the gut mucosa in vaccinated animals, although it could also be examples of the transient clinical signs we have previously seen in vaccinated goats challenged with wild‐type PPRV (Hodgson et al., 2018). It is also possible that there was some effect of CD8^+^ T‐cell depletion on virus excretion, which we did not assess; two recent papers (Enchery et al., 2019; Schulz et al., 2019) have shown detectable viral RNA in ocular swabs in the absence of detectable viraemia. Although we were unfortunately unable to confirm it in our experimental animals, the dose of specific mAb used in our study has previously been shown to eliminate detectable CD8^+^ T cells from the tissues as well as the blood in treated calves (Naessens et al., 1998) and it is a reasonable assumption that a similar clearance occurred in our animals. Our finding suggests that the primary protection in animals vaccinated against PPRV is mediated by antibody and/or CD4^+^ T cells, both of which are elicited by live PPRV vaccines (Hodgson et al., 2018).

It is possible to deplete CD4^+^ cells in the same way as we did here for CD8^+^ cells, given a complement‐fixing anti‐ovine/caprine CD4 of the appropriate isotype (IgM or IgG2 for mouse mAbs). It would clearly be useful and informative to carry out a similar study to that described here but targeting CD4^+^ T cells in vaccinated animals, as this would determine whether protection can be provided by antibody alone, or whether any potential new PPRV vaccine has to also elicit specific CD4^+^ T cells. Given the large amounts of mAb required, such a study does require access to a suitable hybridoma clone, which we did not have at the time of this study. It is difficult to deplete B‐cell populations, which rebound within hours after treatment with a complement‐fixing antibody targeting these cells (Naessens et al., 1998), but the importance of antibody responses could be assessed using passive transfer of immunoglobulins from vaccinated to unvaccinated goats, as has been done, for example, to show that neutralizing antibody alone can protect pigs against porcine reproductive and respiratory syndrome virus (Osorio et al., 2002). It is known that maternal antibody to PPRV is passed to kids in colostrum (Balamurugan et al., 2012), but colostrum also contains significant numbers of maternal leucocytes which are known to be immunologically active (Meganck et al., 2014/and references therein); colostrum has been shown to transfer significantly more protection in cattle than large scale transfusion of maternal immunoglobins (Boccardo et al., 2016). A proper study using transfusion of purified immunoglobins from animals with a good neutralizing titre against PPRV would also be a useful and informative contribution to our understanding of the requirements for protection that any vaccine must fulfil.

The pathogenicity of PPRV varies greatly in the field (Lefevre & Diallo, 1990), variation that has been ascribed to both the virus strain and the host animals (Couacy‐Hymann et al., 2007; Khan et al., 2018). Large scale field surveillance has shown case fatality rates around 40% in India and Pakistan (Abubakar et al., 2018; Bardhan et al., 2017), while case fatality rates in West Africa appear slightly lower (Lefevre & Diallo, 1990). We had available to us PPRV strains of low (e.g. Nigeria/76), intermediate (e.g. Sudan/72, Guinea‐Bissau/91) and high (e.g. Iran/2011, Ghana/78) pathogenicity (in our goats) (Baron et al., 2014) and opted to use an intermediate pathogenicity strain as reflecting an ‘average’ PPRV field strain; it is possible that we may have observed a requirement for CD8^+^ T cells in protection from a higher pathogenicity strain. We also recognize that this study was carried out only 4 weeks after vaccination, and the role(s) played by specific subtypes of immune cell may be different a year or more after vaccination when expansion of memory cells would be required for protection.

The general leucopenia seen during the acute phase of morbillivirus infections has been long noted, particularly for MV infection (e.g. Benjamin & Ward, 1932). There have been only a few studies on the specific effect of MV infection on different cell types: the evidence suggests that the period of overall leucopenia is similar to that seen here for PPRV in goats, lasting about two weeks from the onset of rash in humans infected with MV (Lisse et al., 1998; Okada et al., 2000; Wesley et al., 1978). The extent is generally severe, with loss of 90% of circulating white cells (Okada et al., 2000), and affects at least CD4^+^ and CD8^+^ T cells and B cells (Okada et al., 2000; Wesley et al., 1978); other studies found that the proportions of different cell types remained unchanged during infection (Arneborn & Biberfeld, 1983; Griffin et al., 1986), suggesting that the initial leucopenia in measles also affects all subtypes, as we found for PPRV. Interestingly, recovery of B‐cell numbers was reported as much slower than recovery of T‐cell numbers in MV‐infected humans (Okada et al., 2000; Wesley et al., 1978), which is in line with our observation that numbers of CD21^+^ cells (a marker for B cells) recovered more slowly in PPRV‐infected goats than the different T‐cell types (Figure 1). These findings suggest that the leucopenia seen in animals infected with PPRV is similar to that seen in MV infection. We know from the extensive studies on MV, both in humans and in model systems (particularly the macaque), that MV infection also leads to long‐term immunosuppression (Griffin, 2010; Schneider‐Schaulies & Schneider‐Schaulies, 2009; de Vries et al., 2012), with reduced response to new antigens as well as loss of recall immune responses, leading to the hypothesis that a major effect of measles disease is the destruction of CD4^+^ memory cells in the lymph nodes, leaving survivors more susceptible to other pathogens, even ones to which they were previously immune (de Vries et al., 2012). A similar loss of immune memory was seen in RPV‐infected animals (Yamanouchi et al., 1974), although it was never so extensively studied. If this is also true of PPRV infection, it places even more emphasis on the need to control PPR as a disease of small ruminants, as outbreaks of PPR may be leaving populations of livestock susceptible to diseases that they had previously been vaccinated against, as has clearly been shown for MV infection in humans (Mina et al., 2015, 2019).

## 5 ETHICS STATEMENT

The authors confirm that the ethical policies of the journal, as noted on the journal's author guidelines page, have been adhered to and the appropriate ethical review committee approval was received. Animal studies, the results of which are reported in this manuscript, were carried out in accordance with the Animals (Scientific Procedures) Act 1986 as amended (SI 2012/3029) and approved by the institute Animal Welfare and Ethical Review Board.

## CONFLICTS OF INTEREST

The authors declare that there are no conflicts of interest.

## DATA SHARING

The data that support the findings of this study are available from the corresponding authors upon reasonable request.

## Supporting information

Supplementary Material

## References

[tbed13936-bib-0001] Abubakar, M. , Zahur, A. B. , Naeem, K. , Khan, M. A. , & Qureshi, S. (2018). Field and molecular epidemiology of peste des petits ruminants in Pakistan. Pakistan Journal of Zoology, 50(2), 559–566. 10.17582/journal.pjz/2018.50.2.559.56

[tbed13936-bib-0002] Aguilar, X. F. , Fine, A. E. , Pruvot, M. , Njeumi, F. , Walzer, C. , Kock, R. , & Shiilegdamba, E. (2018). PPR virus threatens wildlife conservation. Science, 363(6411), 165–166. 10.1126/science.aav4096 30309937

[tbed13936-bib-0003] Arneborn, P. , & Biberfeld, G. (1983). T‐lymphocyte subpopulations in relation to immunosuppression in measles and varicella. Infection and Immunity, 39(1), 29–37. 10.1128/IAI.39.1.29-37.1983 6600445 PMC347903

[tbed13936-bib-0004] Balamurugan, V. , Sen, A. , Venkatesan, G. , Rajak, K. K. , Bhanuprakash, V. , & Singh, R. K. (2012). Study on passive immunity: Time of vaccination in kids born to goats vaccinated against Peste des petits ruminants. Virologica Sinica, 27(4), 228–233. 10.1007/s12250-012-3249-6 22899430 PMC8218118

[tbed13936-bib-0005] Bardhan, D. , Kumar, S. , Anandsekaran, G. , Chaudhury, J. K. , Meraj, M. , Singh, R. K. , & De, U. K. (2017). The economic impact of peste des petits ruminants in India. Revue Scientifique Et Technique, Office International Des Epizooties, 36(1), 245–263. 10.20506/rst.36.1.2626 28926011

[tbed13936-bib-0006] Baron, J. , Bin‐Tarif, A. , Herbert, R. , Frost, L. , Taylor, G. , & Baron, M. D. (2014). Early changes in cytokine expression in peste des petits ruminants disease. Veterinary Research, 45(1), 22. 10.1186/1297-9716-45-22 24559207 PMC3941941

[tbed13936-bib-0007] Baron, M. D. , Diallo, A. , Lancelot, R. , & Libeau, G. (2016). Peste des Petits ruminants virus. Advances in Virus Research, 95, 1–42. 10.1016/bs.aivir.2016.02.001 27112279

[tbed13936-bib-0008] Baron, M. D. , Diop, B. , Njeumi, F. , Willett, B. J. , & Bailey, D. (2017). Future research to underpin successful peste des petits ruminants virus (PPRV) eradication. Journal of General Virology, 98(11), 2635–2644. 10.1099/jgv.0.000944 29022862 PMC5845661

[tbed13936-bib-0009] Bassiri, M. , Ahmad, S. , Giavedoni, L. , Jones, L. , Saliki, J. T. , Mebus, C. , & Yilma, T. (1993). Immunological responses of mice and cattle to baculovirus‐expressed F and H proteins of rinderpest virus: Lack of protection in the presence of neutralizing antibody. Journal of Virology, 67(3), 1255–1261. 10.1128/JVI.67.3.1255-1261.1993 8437215 PMC237491

[tbed13936-bib-0010] Benjamin, B. , & Ward, S. M. (1932). Leukocytic response to measles. American Journal of Diseases of Children, 44, 921–963. 10.1001/archpedi.1932.01950120003001

[tbed13936-bib-0011] Boccardo, A. , Belloli, A. , Biffani, S. , Locatelli, V. , Dall'Ara, P. , Filipe, J. , Restelli, I. , Proverbio, D. , & Pravettoni, D. (2016). Intravenous immunoglobulin transfusion in colostrum‐deprived dairy calves. Veterinary Journal, 209, 93–97. 10.1016/j.tvjl.2015.11.015 26831168

[tbed13936-bib-0012] Brinckmann, U. G. , Bankamp, B. , Reich, A. , ter Meulen, V. , & Liebert, U. G. (1991). Efficacy of individual measles virus structural proteins in the protection of rats from measles encephalitis. Journal of General Virology, 72(Pt 10), 2491–2500. 10.1099/0022-1317-72-10-2491 1833505

[tbed13936-bib-0013] Carr, B. V. , Lefevre, E. A. , Windsor, M. A. , Inghese, C. , Gubbins, S. , Prentice, H. , Juleff, N. D. , & Charleston, B. (2013). CD4+ T‐cell responses to foot‐and‐mouth disease virus in vaccinated cattle. Journal of General Virology, 94(Pt 1), 97–107. 10.1099/vir.0.045732-0 23034593 PMC3542717

[tbed13936-bib-0014] Caufour, P. , Rufael, T. , Lamien, C. E. , Lancelot, R. , Kidane, M. , Awel, D. , Sertse, T. , Kwiatek, O. , Libeau, G. , Sahle, M. , Diallo, A. , & Albina, E. (2014). Protective efficacy of a single immunization with capripoxvirus‐vectored recombinant peste des petits ruminants vaccines in presence of pre‐existing immunity. Vaccine, 32(30), 3772–3779. 10.1016/j.vaccine.2014.05.025 24837763

[tbed13936-bib-0015] Chaudhri, G. , Tahiliani, V. , Eldi, P. , & Karupiah, G. (2015). Vaccine‐induced protection against orthopoxvirus infection is mediated through the combined functions of CD4 T cell‐dependent antibody and CD8 T cell responses. Journal of Virology, 89(3), 1889–1899. 10.1128/JVI.02572-14 25428875 PMC4300738

[tbed13936-bib-0016] Cook, J. K. , Jones, B. V. , Ellis, M. M. , Jing, L. , & Cavanagh, D. (1993). Antigenic differentiation of strains of turkey rhinotracheitis virus using monoclonal antibodies. Avian Pathology, 22(2), 257–273. 10.1080/03079459308418919 18671016

[tbed13936-bib-0017] Couacy‐Hymann, E. , Bodjo, C. , Danho, T. , Libeau, G. , & Diallo, A. (2007). Evaluation of the virulence of some strains of peste‐des‐petits‐ruminants virus (PPRV) in experimentally infected West African dwarf goats. Veterinary Journal, 173(1), 178–183. 10.1016/j.tvjl.2005.08.020 16310383

[tbed13936-bib-0018] Davis, W. C. , & Ellis, J. A. (1991). Individual antigens of goats. Veterinary Immunology and Immunopathology, 27(1–3), 121–131. 10.1016/0165-2427(91)90091-p 2021058

[tbed13936-bib-0019] de Vries, R. D. , McQuaid, S. , van Amerongen, G. , Yüksel, S. , Verburgh, R. J. , Osterhaus, A. D. M. E. , Duprex, W. P. , & de Swart, R. L. (2012). Measles immune suppression: Lessons from the macaque model. PLoS Path, 8(8), e1002885. 10.1371/journal.ppat.1002885 PMC3431343 22952446

[tbed13936-bib-0020] Diallo, A. , Minet, C. , Berhe, G. , Goff, C. , Black, D. N. , Fleming, M. , Barrett, T. , Grillet, C. , & Libeau, G. (2002). Goat immune response to capripox vaccine expressing the hemagglutinin protein of peste des petits ruminants. Annals of the New York Academy of Sciences, 969, 88–91. 10.1111/j.1749-6632.2002.tb04356.x 12381569

[tbed13936-bib-0021] Diallo, A. , Taylor, W. P. , Lefevre, P. C. , & Provost, A. (1989). Atténuation d’une souche de virus de la peste des petits ruminants: Candidat pour un vaccin homologue. Revue D'elevage Et De Medecine Veterinaire Des Pays Tropicaux, 42(3), 311–317. 2485537

[tbed13936-bib-0022] Diaz‐Arevalo, D. , Bagramyan, K. , Hong, T. B. , Ito, J. I. , & Kalkum, M. (2011). CD4+ T cells mediate the protective effect of the recombinant Asp f3‐based anti‐aspergillosis vaccine. Infection and Immunity, 79(6), 2257–2266. 10.1128/IAI.01311-10 21422177 PMC3125823

[tbed13936-bib-0023] Elong Ngono, A. , Young, M. P. , Bunz, M. , Xu, Z. , Hattakam, S. , Vizcarra, E. , Regla‐Nava, J. A. , Tang, W. W. , Yamabhai, M. , Wen, J. , & Shresta, S. (2019). CD4+ T cells promote humoral immunity and viral control during Zika virus infection. PLoS Path, 15(1), e1007474. 10.1371/journal.ppat.1007474 PMC6345435 30677097

[tbed13936-bib-0024] Enchery, F. , Hamers, C. , Kwiatek, O. , Gaillardet, D. , Montange, C. , Brunel, H. , Goutebroze, S. , Philippe‐Reversat, C. , Libeau, G. , Hudelet, P. , & Bataille, A. (2019). Development of a PPRV challenge model in goats and its use to assess the efficacy of a PPR vaccine. Vaccine, 37(12), 1667–1673. 10.1016/j.vaccine.2019.01.057 30772071

[tbed13936-bib-0025] Enders, J. F. , Katz, S. L. , Milovanovic, M. V. , & Holloway, A. (1960). Studies on an attenuated measles‐virus vaccine. I. Development and preparations of the vaccine: Technics for assay of effects of vaccination. New England Journal of Medicine, 263, 153–159. 10.1056/NEJM196007282630401 13820246

[tbed13936-bib-0026] Eriksson, K. , McInnes, E. , Ryan, S. , Tonks, P. , McConnell, I. , & Blacklaws, B. (1999). In vivo depletion of CD8+ cells does not affect primary maedi visna virus infection in sheep. Veterinary Immunology and Immunopathology, 70(3–4), 173–187. 10.1016/s0165-2427(99)00061-6 10507360

[tbed13936-bib-0027] Fakri, F. , Bamouh, Z. , Ghzal, F. , Baha, W. , Tadlaoui, K. , Fihri, O. F. , Chen, W. , Bu, Z. , & Elharrak, M. (2018). Comparative evaluation of three capripoxvirus‐vectored peste des petits ruminants vaccines. Virology, 514, 211–215. 10.1016/j.virol.2017.11.015 29197721

[tbed13936-bib-0028] FAO & OIE . (2011). Joint FAO/OIE Committee on Global Rinderpest Eradication: Final Report May 2011. FAO & OIE. https://www.oie.int/doc/ged/D10943.PDF.

[tbed13936-bib-0029] FAO & OIE. (2015). Global control and eradication of peste des petits ruminants. FAO & OIE. https://www.oie.int/eng/PPR2015/doc/PPR‐Advocacy‐EN.pdf.

[tbed13936-bib-0030] Fischer, L. , Tronel, J. P. , Pardo‐David, C. , Tanner, P. , Colombet, G. , Minke, J. , & Audonnet, J. C. (2002). Vaccination of puppies born to immune dams with a canine adenovirus‐based vaccine protects against a canine distemper virus challenge. Vaccine, 20(29–30), 3485–3497. 10.1016/S0264-410X(02)00344-4 12297394

[tbed13936-bib-0031] Fooks, A. R. , Jeevarajah, D. , Lee, J. , ter Meulen, V. , Clegg, J. C. , Warnes, A. , Niewiesk, S. , & Stephenson, J. R. (1998). Oral or parenteral administration of replication‐deficient adenoviruses expressing the measles virus haemagglutinin and fusion proteins: Protective immune responses in rodents. Journal of General Virology, 79, 1027–1031. 10.1099/0022-1317-79-5-1027 9603317

[tbed13936-bib-0032] Good, R. A. , & Zak, S. J. (1956). Disturbances in gamma globulin synthesis as experiments of nature. Pediatrics, 18(1), 109–149. 13335330

[tbed13936-bib-0033] Griffin, D. E. (2010). Measles virus‐induced suppression of immune responses. Immunological Reviews, 236, 176–189. 10.1111/j.1600-065X.2010.00925.x 20636817 PMC2908915

[tbed13936-bib-0034] Griffin, D. E. , Moench, T. R. , Johnson, R. T. , Lindo de Soriano, I. , & Vaisberg, A. (1986). Peripheral blood mononuclear cells during natural measles virus infection: Cell surface phenotypes and evidence for activation. Clinical Immunology and Immunopathology, 40(2), 305–312. 10.1016/0090-1229(86)90035-8 3087668

[tbed13936-bib-0035] Herbert, R. , Baron, J. , Batten, C. , Baron, M. , & Taylor, G. (2014). Recombinant adenovirus expressing the haemagglutinin of peste des petits ruminants virus (PPRV) protects goats against challenge with pathogenic virus; a DIVA vaccine for PPR. Veterinary Research, 45(1), 24. 10.1186/1297-9716-45-24 24568545 PMC3941483

[tbed13936-bib-0036] Hodgson, S. , Moffat, K. , Hill, H. , Flannery, J. T. , Graham, S. P. , Baron, M. D. , & Darpel, K. E. (2018). Comparison of the immunogenicities and cross‐lineage efficacies of live attenuated peste des petits ruminants virus vaccines PPRV/Nigeria/75/1 and PPRV/Sungri/96. Journal of Virology, 92(24), 10.1128/JVI.01471-18 PMC6258957 30258008

[tbed13936-bib-0037] Holm, S. (1979). A simple sequentially rejective multiple test procedure. Scandinavian Journal of Statistics, 6, 65–70.

[tbed13936-bib-0038] Holzer, B. , Hodgson, S. , Logan, N. , Willett, B. , & Baron, M. D. (2016). Protection of cattle against rinderpest by vaccination with wild‐type but not attenuated strains of peste des petits ruminants virus. Journal of Virology, 90(10), 5152–5162. 10.1128/JVI.00040-16 26984722 PMC4859729

[tbed13936-bib-0039] Hope, J. C. , Sopp, P. , & Howard, C. J. (2002). NK‐like CD8(+) cells in immunologically naive neonatal calves that respond to dendritic cells infected with Mycobacterium bovis BCG. Journal of Leukocyte Biology, 71(2), 184–194. 11818438

[tbed13936-bib-0040] Juleff, N. , Windsor, M. , Lefevre, E. A. , Gubbins, S. , Hamblin, P. , Reid, E. , & Charleston, B. (2009). Foot‐and‐mouth disease virus can induce a specific and rapid CD4+ T‐cell‐independent neutralizing and isotype class‐switched antibody response in naive cattle. Journal of Virology, 83(8), 3626–3636. 10.1128/JVI.02613-08 19176618 PMC2663275

[tbed13936-bib-0041] Karanu, F. N. , McGuire, T. C. , Davis, W. C. , Besser, T. E. , & Jasmer, D. P. (1997). CD4+ T lymphocytes contribute to protective immunity induced in sheep and goats by *Haemonchus contortus* gut antigens. Parasite Immunology, 19(10), 435–445. 9372511 10.1046/j.1365-3024.1997.d01-149.x

[tbed13936-bib-0042] Khan, A. , Saleemi, M. K. , Ali, F. , Abubakar, M. , Hussain, R. , Abbas, R. Z. , & Khan, I. A. (2018). Pathophysiology of peste des petits ruminants in sheep (Dorper & Kajli) and goats (Boer & Beetal). Microbial Pathogenesis, 117, 139–147. 10.1016/j.micpath.2018.02.009 29427710

[tbed13936-bib-0043] Lefevre, P. C. , & Diallo, A. (1990). Peste des petits ruminants. Revue Scientifique Et Technique, Office International Des Epizooties, 9(4), 935–981. 10.20506/rst.9.4.532 2132714

[tbed13936-bib-0044] Lin, W. H. , Pan, C. H. , Adams, R. J. , Laube, B. L. , & Griffin, D. E. (2014). Vaccine‐induced measles virus‐specific T cells do not prevent infection or disease but facilitate subsequent clearance of viral RNA. MBio, 5(2), e01047. 10.1128/mBio.01047-14 24736226 PMC3993862

[tbed13936-bib-0045] Lisse, I. , Samb, B. , Whittle, H. , Jensen, H. , Soumare, M. , Simondon, F. , & Aaby, P. (1998). Acute and long‐term changes in T‐lymphocyte subsets in response to clinical and subclinical measles. A community study from rural Senegal. Scandinavian Journal of Infectious Diseases, 30(1), 17–21. 10.1080/003655498750002240 9670353

[tbed13936-bib-0046] MacHugh, N. D. , Bensaid, A. , Howard, C. J. , Davis, W. C. , & Morrison, W. I. (1991). Analysis of the reactivity of anti‐bovine CD8 monoclonal antibodies with cloned T cell lines and mouse L‐cells transfected with bovine CD8. Veterinary Immunology and Immunopathology, 27(1–3), 169–172. 10.1016/0165-2427(91)90096-u 1902341

[tbed13936-bib-0047] MacHugh, N. D. , & Sopp, P. (1991). Individual antigens of cattle. Bovine CD8 (BoCD8). Veterinary Immunology and Immunopathology, 27(1–3), 65–69. 10.1016/0165-2427(91)90081-M 1708557

[tbed13936-bib-0048] Mackay, C. R. , Hein, W. R. , Brown, M. H. , & Matzinger, P. (1988). Unusual expression of CD2 in sheep: Implications for T cell interactions. European Journal of Immunology, 18(11), 1681–1688. 10.1002/eji.1830181105 2462499

[tbed13936-bib-0049] McClure, S. J. , & Hein, W. R. (1989). Functional characteristics of 197+ CD4‐ CD8‐ sheep T lymphocytes: Expansion and differentiation of peripheral T cells. Immunology and Cell Biology, 67(Pt 4), 223–231. 10.1038/icb.1989.34 2670753

[tbed13936-bib-0050] Meganck, V. , Goddeeris, B. M. , Stuyven, E. , Piepers, S. , Cox, E. , & Opsomer, G. (2014). Development of a method for isolating bovine colostrum mononuclear leukocytes for phenotyping and functional studies. Veterinary Journal, 200(2), 294–298. 10.1016/j.tvjl.2014.02.021 24679458

[tbed13936-bib-0051] Mina, M. J. , Kula, T. , Leng, Y. , Li, M. , de Vries, R. D. , Knip, M. , Siljander, H. , Rewers, M. , Choy, D. F. , Wilson, M. S. , Larman, H. B. , Nelson, A. N. , Griffin, D. E. , de Swart, R. L. , & Elledge, S. J. (2019). Measles virus infection diminishes preexisting antibodies that offer protection from other pathogens. Science, 366(6465), 599–606. 10.1126/science.aay6485 31672891 PMC8590458

[tbed13936-bib-0052] Mina, M. J. , Metcalf, C. J. , de Swart, R. L. , Osterhaus, A. D. , & Grenfell, B. T. (2015). Long‐term measles‐induced immunomodulation increases overall childhood infectious disease mortality. Science, 348(6235), 694–699. 10.1126/science.aaa3662 25954009 PMC4823017

[tbed13936-bib-0053] Moss, W. J. (2017). Measles. The Lancet, 390(10111), 2490–2502. 10.1016/S0140-6736(17)31463-0 28673424

[tbed13936-bib-0054] Naessens, J. , Newson, J. , McHugh, N. , Howard, C. J. , Parsons, K. , & Jones, B. (1990). Characterization of a bovine leucocyte differentiation antigen of 145,000 MW restricted to B lymphocytes. Immunology, 69(4), 525–530. 2185984 PMC1385623

[tbed13936-bib-0055] Naessens, J. , Scheerlinck, J. P. , De Buysscher, E. V. , Kennedy, D. , & Sileghem, M. (1998). Effective in vivo depletion of T cell subpopulations and loss of memory cells in cattle using mouse monoclonal antibodies. Veterinary Immunology and Immunopathology, 64(3), 219–234. 10.1016/s0165-2427(98)00138-x 9730218

[tbed13936-bib-0056] Okada, H. , Kobune, F. , Sato, T. A. , Kohama, T. , Takeuchi, Y. , Abe, T. , Takayama, N. , Tsuchiya, T. , & Tashiro, M. (2000). Extensive lymphopenia due to apoptosis of uninfected lymphocytes in acute measles patients. Archives of Virology, 145(5), 905–920. 10.1007/s007050050683 10881678

[tbed13936-bib-0057] Osorio, F. A. , Galeota, J. A. , Nelson, E. , Brodersen, B. , Doster, A. , Wills, R. , Zuckermann, F. , & Laegreid, W. W. (2002). Passive transfer of virus‐specific antibodies confers protection against reproductive failure induced by a virulent strain of porcine reproductive and respiratory syndrome virus and establishes sterilizing immunity. Virology, 302(1), 9–20. 10.1006/viro.2002.1612 12429512

[tbed13936-bib-0058] Pena, M. T. , Miller, J. E. , & Horohov, D. W. (2006). Effect of CD4+ T lymphocyte depletion on resistance of Gulf Coast native lambs to *Haemonchus contortus* infection. Veterinary Parasitology, 138(3–4), 240–246. 10.1016/j.vetpar.2005.12.026 16516389

[tbed13936-bib-0059] Plowright, W. , & Ferris, R. D. (1962). Studies with rinderpest virus in tissue culture. The use of attenuated culture virus as a vaccine for cattle. Research in Veterinary Science, 3, 172–182. 10.1016/S0034-5288(18)34916-6

[tbed13936-bib-0060] Qin, J. , Huang, H. , Ruan, Y. , Hou, X. , Yang, S. , Wang, C. , Huang, G. , Wang, T. , Feng, N. A. , Gao, Y. , & Xia, X. (2012). A novel recombinant Peste des petits ruminants‐canine adenovirus vaccine elicits long‐lasting neutralizing antibody response against PPR in goats. PLoS One, 7(5), e37170. 10.1371/journal.pone.0037170 22623990 PMC3356378

[tbed13936-bib-0061] Rojas, J. M. , Moreno, H. , Valcarcel, F. , Pena, L. , Sevilla, N. , & Martin, V. (2014). Vaccination with recombinant adenoviruses expressing the peste des petits ruminants virus F or H proteins overcomes viral immunosuppression and induces protective immunity against PPRV challenge in sheep. PLoS One, 9(7), e101226. 10.1371/journal.pone.0101226 25013961 PMC4094465

[tbed13936-bib-0062] Sacchini, F. , Naessens, J. , Awino, E. , Heller, M. , Hlinak, A. , Haider, W. , Sterner‐Kock, A. , & Jores, J. (2011). A minor role of CD4+ T lymphocytes in the control of a primary infection of cattle with *Mycoplasma mycoides* subsp. *mycoides* . Veterinary Research, 42, 77. 10.1186/1297-9716-42-77 21663697 PMC3148206

[tbed13936-bib-0063] Schneider‐Schaulies, S. , & Schneider‐Schaulies, J. (2009). Measles virus‐induced immunosuppression. Current Topics in Microbiology and Immunology, 330, 243–269. 10.1007/978-3-540-70617-5_12 19203113

[tbed13936-bib-0064] Schulz, C. , Fast, C. , Wernery, U. , Kinne, J. , Joseph, S. , Schlottau, K. , Jenckel, M. , Höper, D. , Patteril, N. A. G. , Syriac, G. , Hoffmann, B. , & Beer, M. (2019). Camelids and cattle are dead‐end hosts for peste‐des‐petits‐ruminants virus. Viruses, 11(12), 10.3390/v11121133 PMC6950723 31817946

[tbed13936-bib-0065] Sileghem, M. , & Naessens, J. (1995). Are CD8 T cells involved in control of African trypanosomiasis in a natural host environment? European Journal of Immunology, 25(7), 1965–1971. 10.1002/eji.1830250725 7621872

[tbed13936-bib-0066] Sullivan, N. J. , Hensley, L. , Asiedu, C. , Geisbert, T. W. , Stanley, D. , Johnson, J. , Honko, A. , Olinger, G. , Bailey, M. , Geisbert, J. B. , Reimann, K. A. , Bao, S. , Rao, S. , Roederer, M. , Jahrling, P. B. , Koup, R. A. , & Nabel, G. J. (2011). CD8+ cellular immunity mediates rAd5 vaccine protection against Ebola virus infection of nonhuman primates. Nature Medicine, 17(9), 1128–1131. 10.1038/nm.2447 21857654

[tbed13936-bib-0067] Taylor, G. , Thomas, L. H. , Wyld, S. G. , Furze, J. , Sopp, P. , & Howard, C. J. (1995). Role of T‐lymphocyte subsets in recovery from respiratory syncytial virus infection in calves. Journal of Virology, 69(11), 6658–6664. 10.1128/JVI.69.11.6658-6664.1995 7474075 PMC189575

[tbed13936-bib-0068] Taylor, J. , Pincus, S. , Tartaglia, J. , Richardson, C. , Alkhatib, G. , Briedis, D. , Appel, M. , Norton, E. , & Paoletti, E. (1991). Vaccinia virus recombinants expressing either the measles virus fusion or hemagglutinin glycoprotein protect dogs against canine distemper virus challenge. Journal of Virology, 65, 4263–4274. 10.1128/JVI.65.8.4263-4274.1991 1830113 PMC248864

[tbed13936-bib-0069] Thomas, L. H. , Cook, R. S. , Howard, C. J. , Gaddum, R. M. , & Taylor, G. (1996). Influence of selective T‐lymphocyte depletion on the lung pathology of gnotobiotic calves and the distribution of different T‐lymphocyte subsets following challenge with bovine respiratory syncytial virus. Research in Veterinary Science, 61(1), 38–44. 10.1016/s0034-5288(96)90108-3 8819192

[tbed13936-bib-0070] Valkenburg, S. A. , Li, O. T. W. , Li, A. , Bull, M. , Waldmann, T. A. , Perera, L. P. , Peiris, M. , & Poon, L. L. M. (2018). Protection by universal influenza vaccine is mediated by memory CD4 T cells. Vaccine, 36(29), 4198–4206. 10.1016/j.vaccine.2018.06.007 29887326 PMC8127355

[tbed13936-bib-0071] Wang, S. , Goguen, J. D. , Li, F. , & Lu, S. (2011). Involvement of CD8+ T cell‐mediated immune responses in LcrV DNA vaccine induced protection against lethal *Yersinia pestis* challenge. Vaccine, 29(39), 6802–6809. 10.1016/j.vaccine.2010.12.062 21199697 PMC3079781

[tbed13936-bib-0072] Wang, Y. , Liu, G. , Chen, Z. , Li, C. , Shi, L. , Li, W. , Huang, H. , Tao, C. , Cheng, C. , Xu, B. , & Li, G. (2013). Recombinant adenovirus expressing F and H fusion proteins of peste des petits ruminants virus induces both humoral and cell‐mediated immune responses in goats. Veterinary Immunology and Immunopathology, 154(1–2), 1–7. 10.1016/j.vetimm.2013.05.002 23707075

[tbed13936-bib-0073] Wesley, A. , Coovadia, H. M. , & Henderson, L. (1978). Immunological recovery after measles. Clinical and Experimental Immunology, 32(3), 540–544. 688697 PMC1541324

[tbed13936-bib-0074] Yamanouchi, K. , Fukuda, A. , Kobune, F. , Yoshikawa, Y. , & Chino, F. (1974). Pathogenesis of rinderpest virus infection in rabbits. II. Effect of rinderpest virus on the immune functions of rabbits. Infection and Immunity, 9(2), 206–211. 4593340 10.1128/iai.9.2.206-211.1974 PMC414788

