# Supplementary material for: Depletion of CD8+ T cells from vaccinated goats does not affect protection from challenge with wild‐type peste des petits ruminants virus
Source: Transbound Emerg Dis. 2020 Dec 20;68(6):3320–34. doi: 10.1111/tbed.13936 (PMC9291567; doi:10.1111/tbed.13936)
Supplement: Supplementary file 1 — Supplementary Material [file TBED-68-3320-s001.pdf]

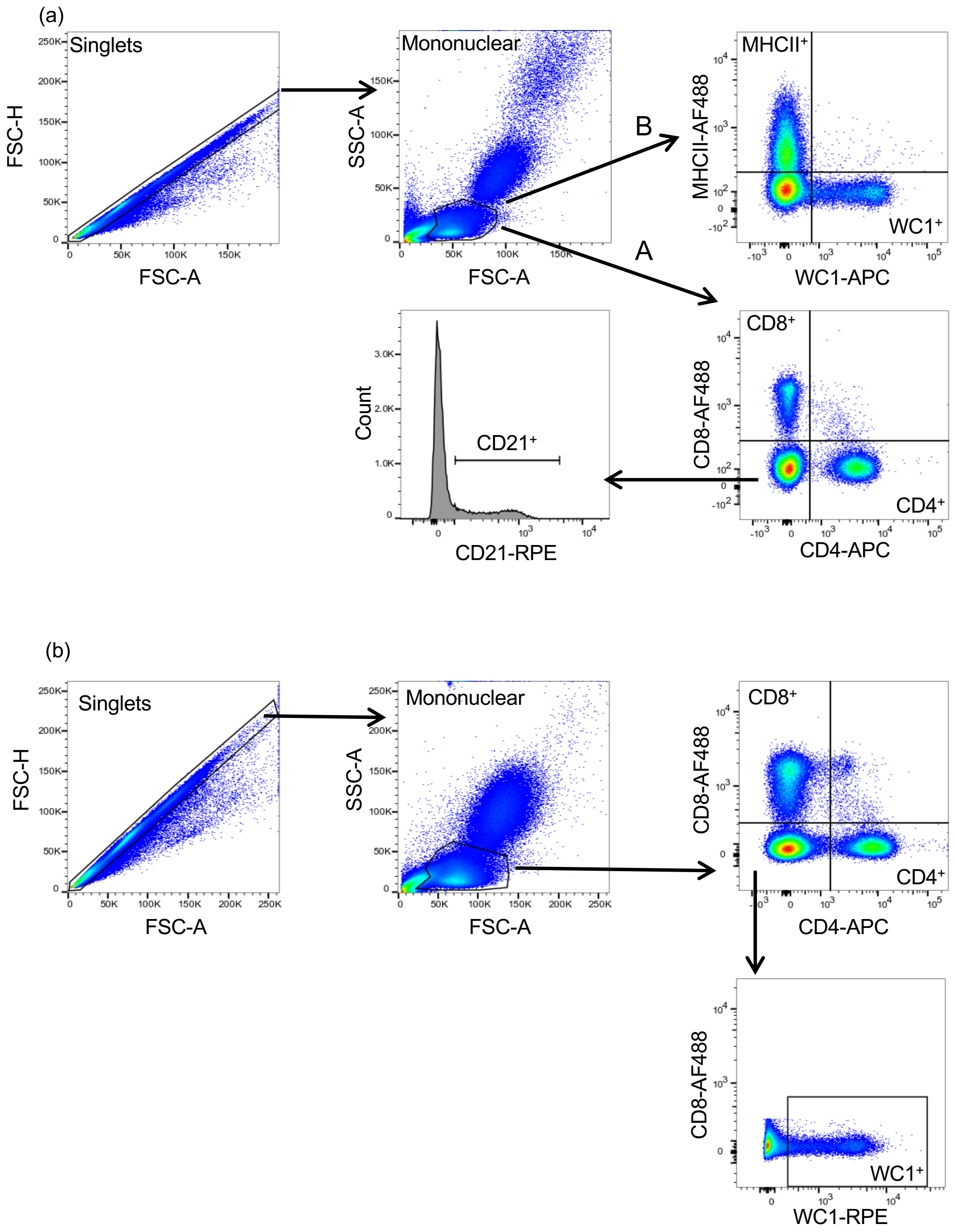

Figure S1 Gating strategies used in these studies
(a) Gating strategies used for whole blood labelling in infected animals. Blood samples were labelled with either of two antibody cocktails, A or B, as described in Methods. An enhanced lymphocyte gate (Mononuclear) was used to ensure capture of antigen presenting cells. The different cell types were identified as shown. (b) Gating strategy used for whole blood labelling in depletion studies. The strategy here was similar to (a), except that only one antibody cocktail was used and three main cell types identified.

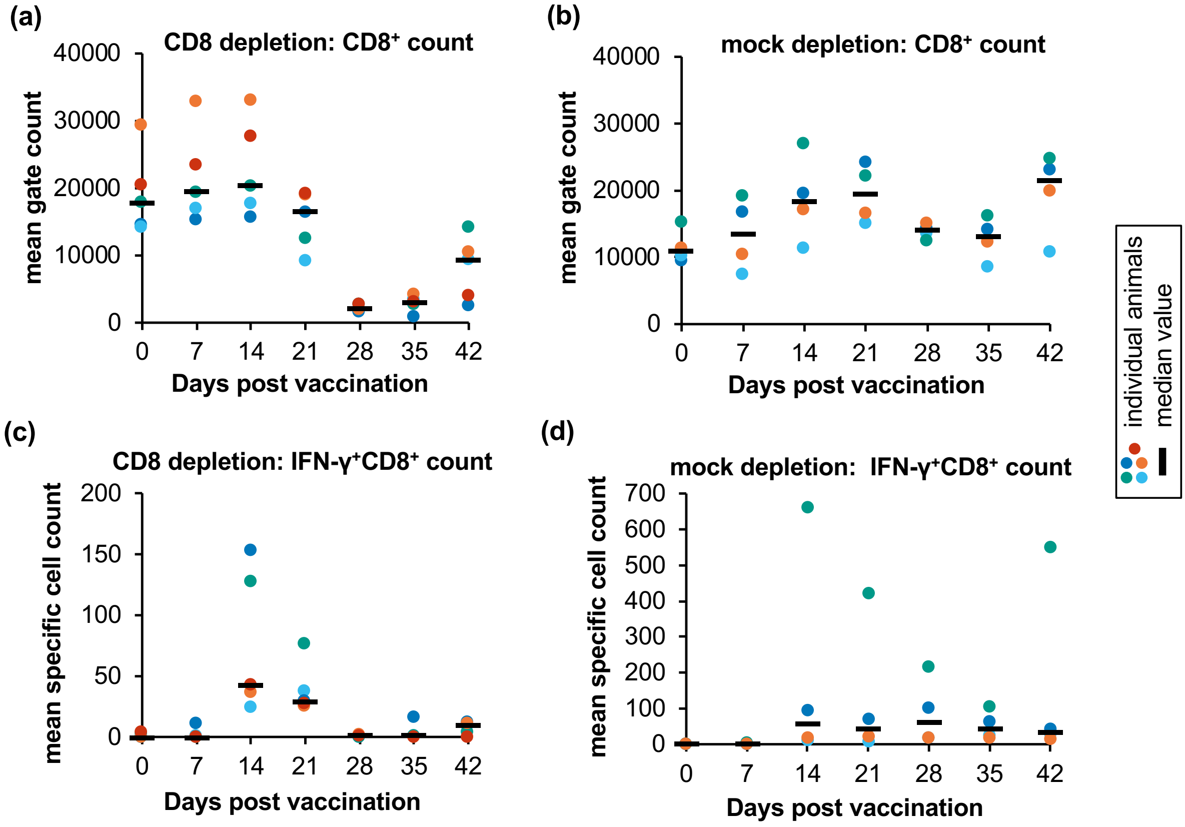

Figure S2 Flow cytometry data measurement of $CD8^+$ T cells and PPRV-specific $CD8^+$ T cells (a, b) The mean number of events in the $CD8^+$ T cell gate for samples of PBMCs prepared from the animals in (a) the CD8 depletion group and (b) the mock depletion group on the given days; colour dots: individual animals; black bar: the median for the set of animals. (c, d) The number of specific events in the $CD8^+IFN\text{-}\gamma^+$ gate, averaged over the responses to N75 and S96 vaccine viruses, where specific events were (number of events after stimulation with virus) - (number of events after stimulation with control preparation), for the animals in (c) the CD8 depletion group and (d) the mock depletion group; colour dots: individual animals; black bar: the median for the set of animals.

Example flow cytometry data is shown in Figure S3

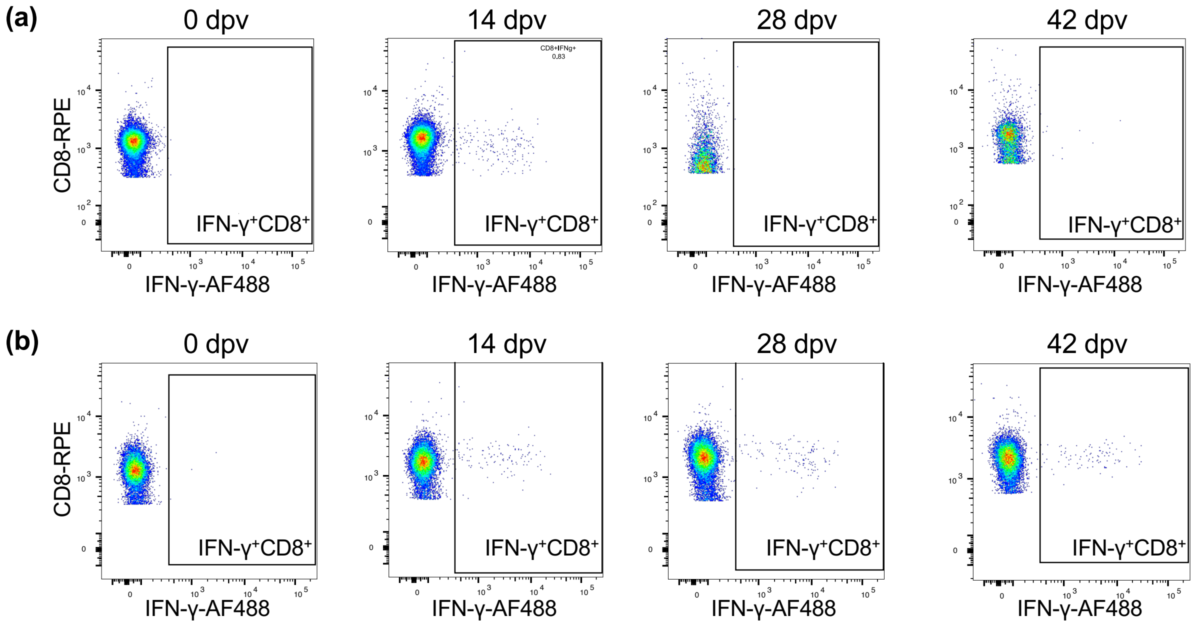

Figure S3 Sample flow cytometry data from studies of PPRV-specific CD8$^+$ T cells during the depletion-challenge experiment. PBMCs from experimental animals were stimulated with PPRV virus or viral antigen and IFN-γ$^+$CD8$^+$ T cells identified by immunofluorescent labelling and flow cytometry as described in Materials and Methods. Shown are sample data from PBMCs purified before vaccination (0 dpv), after vaccination (14 dpv), during the peak effect of the depletion (28 dpv) and at the end of the study (42 dpv) and stimulated with S96 vaccine virus: (a) PBMCS from one animal from the CD8 depletion group and (b) PBMCs from one animal from the mock depletion group.
